# Mushrooms as future generation healthy foods

**DOI:** 10.3389/fnut.2022.1050099

**Published:** 2022-12-06

**Authors:** V. Bell, C. R. P. G. Silva, J. Guina, T. H. Fernandes

**Affiliations:** ^1^Faculty of Pharmacy, University of Coimbra, Pólo das Ciências da Saúde, Coimbra, Portugal; ^2^Department of Health and Social Care, School of Health and Care Management, Arden University, Coventry, United Kingdom; ^3^Instituto Superior de Estudos Universitários de Nampula (ISEUNA), Universidade a Politécnica, Nampula, Mozambique; ^4^CIISA—Centre for Interdisciplinary Research in Animal Health, Faculty of Veterinary Medicine, Associate Laboratory for Animal and Veterinary Sciences (AL4AnimalS), University of Lisbon, Lisbon, Portugal; ^5^Centro de Estudos Interdisciplinares Lurio (CEIL), Lúrio University, Nampula, Mozambique

**Keywords:** fungi nourishment, bioactive elements, healthcare prevention, functional foods, pharmanutrients

## Abstract

The potential of edible mushrooms as an unexploited treasure trove, although rarely included in known food guidelines, is highlighted. Their role in shielding people against the side effects of an unhealthy stylish diet is reviewed. Mushrooms complement the human diet with various bioactive molecules not identified or deficient in foodstuffs of plant and animal sources, being considered a functional food for the prevention of several human diseases. Mushrooms have been widely used as medicinal products for more than 2,000 years, but globally the potential field of use of wild mushrooms has been untapped. There is a broad range of edible mushrooms which remain poorly identified or even unreported which is a valuable pool as sources of bioactive compounds for biopharma utilization and new dietary supplements. Some unique elements of mushrooms and their role in preventative healthcare are emphasized, through their positive impact on the immune system. The potential of mushrooms as antiviral, anti-inflammatory, anti-neoplastic, and other health concerns is discussed. Mushrooms incorporate top sources of non-digestible oligosaccharides, and ergothioneine, which humans are unable to synthesize, the later a unique antioxidant, cytoprotective, and anti-inflammatory element, with therapeutic potential, approved by world food agencies. The prebiotic activity of mushrooms beneficially affects gut homeostasis performance and the balance of gut microbiota is enhanced. Several recent studies on neurological impact and contribution to the growth of nerve and brain cells are mentioned. Indeed, mushrooms as functional foods' nutraceuticals are presently regarded as next-generation foods, supporting health and wellness, and are promising prophylactic or therapeutic agents.

## Introduction

The global food system is very complex and influenced by many different inputs, including farming, economics, politics, environment, transport, storage, and consumers, and it must entail long-term dimensions of sustainability. A healthy, resilient, and sustainable food system supporting a growing poor population in a changing environment, aggravated by pandemics and wars, requires structural changes and innovations (1).

Agriculture and food security are the basis to support human health and dietary patterns by improving agricultural diversity and providing the basis for a balanced healthy diet. The occurrence of both under-nutrition and over-nutrition in the same community, even in the same household, is common probably due to unsound diets, economic globalization, or diseases (2).

Few food and nutrient guidelines were established by a handful of agencies to provide consumers and health practitioners with evidence-based recommendations on nutrient and dietary intakes associated with low risk of nutritional deficiencies and diet-related chronic diseases. However, there is little health research on diet quality based on the standard diet consumed and universal micronutrient supplementation (3, 4).

Mushrooms are edible fungus and have been widely used as medicinal products in China, Japan, and Korea. In other countries, only in the past few decades, special attention has been given to dietary supplements as sources to improve health and wellness. The nutritional role of mushroom products, as indirect probiotics, as direct prebiotics, or as both (synbiotics), is justified by their influence on the inflammation process and the gut microbiota through their contents of β-glucans, enzymes, and secondary metabolites (5).

Gut microbiota have different and specific profiles from different regions and populations, which need to be studied in order to match and determine their nutrient requirements as well as the widespread anti-biotic-resistant infections by its over- or misuse (6).

Several fungi bioactive compounds are not found or deficient in human food items of plant and animal origin, are known to support good health and wellbeing and are used as prophylactics for various human diseases. Mushrooms are now recognized as a source of nutraceuticals in nutrient balancing, strengthening the human immune system, enhancing natural body resistance, and lowering proneness to disease (7).

In this review, we underline the potential of edible mushrooms, as novel food sources and surprisingly rarely included in dietary guidelines, as an unexploited gold mine and one of the greatest untapped food resources to provide income for growing and poorer populations, and a role in shielding people against the side effects of an unhealthy stylish diet.

## Mushrooms

Fungi are eukaryotic organisms that comprise microorganisms such as yeasts and molds, as well as mushrooms and fungi producing macrostructures yielding spores. Mushrooms as macro-fungi are the root of a multitude of compelling secondary metabolites produced in the portion of soil found adjacent to plant roots as self-defense as a response to biotic or non-living factors of stress (8).

The fungus mycelium creates an intercellular network growing between the endodermis single layer of cells bordering the cortex of a plant's root, but not penetrating them, sharing nutrients and water between them. Interaction or symbiosis, as a function of resource allocation, emerges as one of the essential characteristics of life, alongside metabolism and reproduction (9).

The mushroom supplies the plant with water and minerals (e.g., phosphorus and nitrogen) taken from the soil, whereas the plant provides fungi with carbon substrates and energy derived from photosynthesis (10). Mushrooms are indispensable to human life and essential for the environment as major decomposers of organic matter namely in forests and recyclers in nature ecosystems.

Mycorrhizae, known as root fungi, benefit from mutual dependence symbiotic associations entrenched between certain soil fungi and most vascular plants. Ectomycorrhizal fungi (Ascomycetes and Basidiomycetes) at root tips protect plants against pathogens, improve nutrient arrest, remediate heavy metal contamination, and avoid extreme soil pollution (11).

Phenotype-based approaches have been used to establish mushroom diversity. The global distribution of mushrooms has not been yet completely surveyed, identified, and classified under the latest protocols and technological improvements that genome sequencing offers for promising alternatives to DNA barcoding (12).

Mushrooms engage central roles in dynamic and complex communities of plant, animal, and microorganisms, decaying dead humus and interacting with evergreens, being key controllers of carbon and nutrient cycling for the maintenance of biodiversity.

Truffles, underground macro-fungi from arid- or semi-arid ecosystems, are expensive food commodities, greatly appreciated for both their culinary and medicinal properties and for enhancing the capacity of their host plant roots to resist dry spells (13).

They have been used since the Paleolithic period (ca. 2.5 million years ago to 10,000 B.C.), where their application has been historically related to spiritualism. Globally, and despite a lot of speculation, there are some 3–6 million species of fungi, but only some 3–8% have been accurately and precisely identified (14).

Only some 25 species have been perceived as edible and intensively cultivated (15), while only few species are the most produced and consumed edible mushroom worldwide: *Agaricus bisporus* (white mushroom, 22%), followed by *Lentinus edodes* (shiitake, 19%), *Pleurotus* spp. (oyster mushroom, 15%), and *Flammulina velutipes* (enoki mushroom, 11%) (16).

With the application of a collection of new molecular methods to search for biomarkers in the genome and proteome, it is now possible to identify mushrooms previously considered non-existent and detail their low-molecular-weight (e.g., glycosphingolipids, biochrome quinones, and polyphenolic isoflavones) (17) and high-molecular-weight compounds (e.g., different glucans, glycopeptides, and ribonucleoprotein complexes) (18).

Biochemical and nutrient composition of the main industrialized mushrooms is well-known; however, for many wild and under-exploited edible fungi, detailed nutritive data are limited or even non-existent. This is problematic in low-income settings where they are collected for food, as income, and constitute a valuable resource for food security.

**Figure 1:**
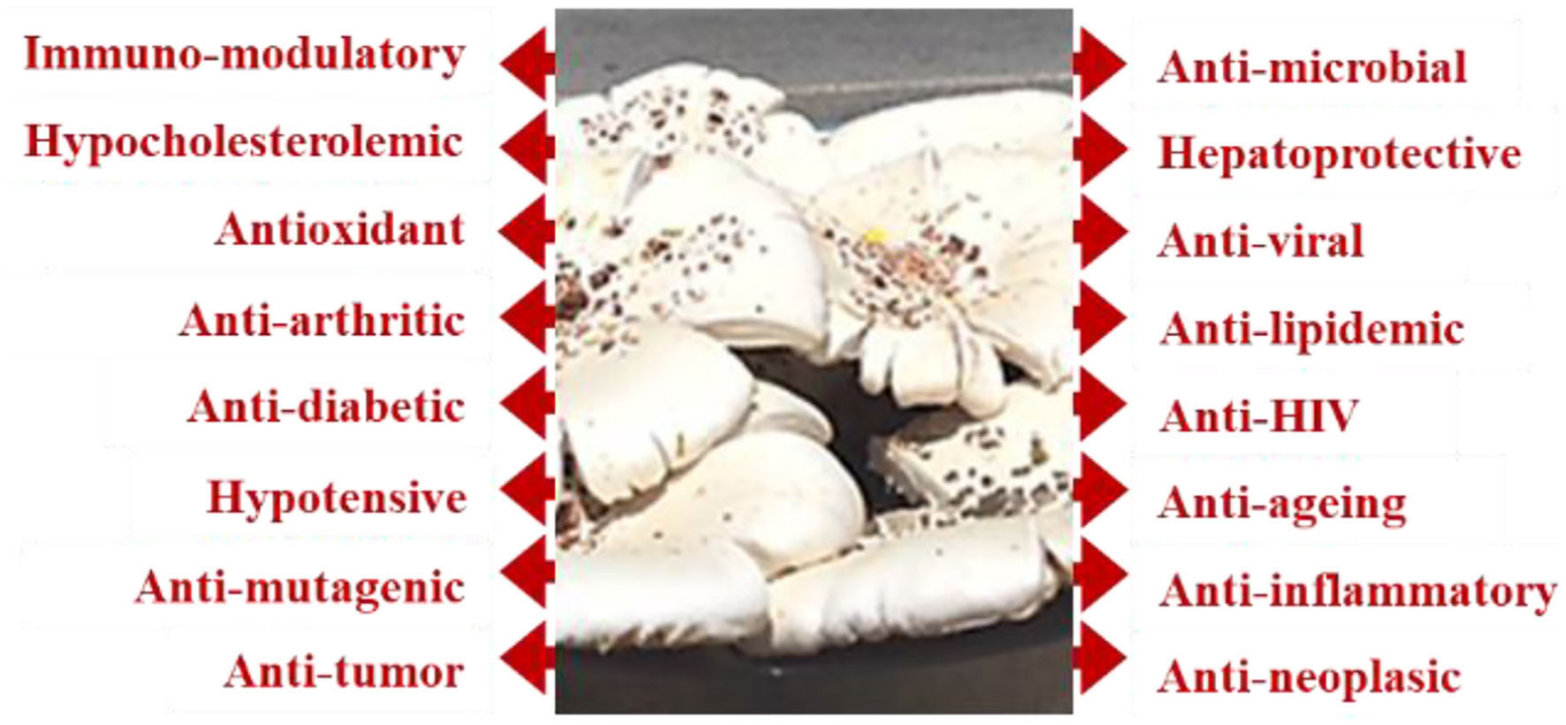
Multiple roles of mushrooms in several dysfunctions and health concerns.

Many research studies (19) have determined the safeguarding health outcomes of wholesome mushrooms to protect or treat various chronic diseases (Figure 1).

Most edible mushrooms, like most food items, have a limited probability of surplus dosage or toxicity and, along with negligible side effects, are perfect candidates for developing novel dietary supplements and therapeutics (20).

Here, up-to-date information is given on the current and factual knowledge on edible mushroom composition, antioxidants, and bioactive elements, enhancing their use in some disorders, and for innovative biotechnological, medicinal, and ecological applications (21). Their application in alternative or complementary folk or traditional medicine is out of the present scope.

## Nutritive value of mushrooms

Many different mushroom species have diversified compositions and nutritional values (22). Common mushrooms provide micronutrients and minimal energy being outstanding suppliers of crude protein, several essential minerals, complex polysaccharides, fat free but with essential unsaturated fatty acids (>75%), vitamins B (B2 riboflavin, B9 folate, B1 thiamine, B5 pantothenic acid, and B3 niacin), and secondary metabolites (23).

Presently, there is growing attention on using mushrooms in the development of natural functional dietary supplements and biotherapeutics, as they may modulate the immune system and act as anti-inflammatory through their high content of antioxidants (24, 25).

For millennia, humans picked and ingested mushrooms for their taste and curative properties, unaware of their nutritive value. Raw fresh mushrooms contain a high concentration of water (85–95%) influencing the specific heat, a property of food materials needed for analysis and design processes involving heat transfer.

Vegans refrain from consuming animal products and may lack vitamin B12 responsible to maintain the myelin surrounding nerve cells, mental ability, red blood cell formation, and the breaking down of fatty and amino acids to produce energy. Vitamin B12 is generally low across most varieties of mushrooms and completely absent from plant sources and has a close relationship with folate, both depending on one another to work properly. Nevertheless, shiitake mushrooms contain the highest amount of vitamin B12 at 5.6 micrograms per 100 g DM (26).

## Bioactive molecules of mushrooms

One of the great assets of fungi and yeasts is their unsophistication. As a eukaryotic organism, with larger and more complex ribosomal protein subunits, its cell biology often relies on fewer factors and fewer complex regulations (27).

The prevailing and vast list of secondary metabolites in mushrooms (e.g., lectins, phenolic carboxylic acids, coenzymes, triterpenoids, and among many other bioactive compounds) may oversee the activation of cellular phagocytes, cytotoxic T cells, and anti-body-mediated immunity, consequently expanding protection to disorders (4).

Mushrooms' performance on intestinal health is *via* specific bioactive molecules, namely oligo- and polysaccharide β-glucans (PSP—polysaccharide peptide or PSK—polysaccharide-krestin) and nucleotides (4). Most of these bioactive compounds function as biological response modifiers (BRM) which are immunomodulation substances that stimulate the body's response to infection and disease (28).

Mushrooms as heterotrophic organisms are unable to perform photosynthesis. They reproduce sexually or asexually, producing special cells called spores, with the potential to produce one billion offspring in a single day. They are saprophytes as they, through their enzymes, break down and absorb complex organic compounds from various environmental matrices, as they are unable to synthesize their own organic matter (29).

Mushrooms contain various bioactive flavonoids and over 30 polyphenolic compounds which act as successful antioxidants rooted in their outstanding capacity to scavenge free radicals and act as reducing agents (30). The abundance of these fungi's small organic molecules exhibits an effective repercussion on the immune system of the human host consumer (31).

Lignocellulose biomass waste is the most significant natural complex biopolymer on earth, and its structure of cellulose, hemicellulose, and lignin, being so intricate, affects its biodegradation and rate-limiting steps in the global carbon cycle, atmosphere, oceans, fossil fuels, land, and forests (32). Most species of mushrooms synthesize numerous fungal enzymes that play important functions in various applications for human, plant, and environment systems (33, 34).

The endless list of mushroom enzymes comprises ligninolytic enzymes (e.g., laccase and lignin peroxidase), hemicellulases (e.g., degrading galactans, xylans, mannans, and arabans), acid protease, β-glucanases, β-glucosidase, esterases, ribonucleases, and many others (35). These mushroom exoenzymes are major decomposing agents of utmost organic matter, biodegrading larger complex molecules, and releasing smaller fragment elements useful as nutrients or energy (36).

Woodland native *Hericium erinaceus* fruiting bodies were reported to have cytoprotective effects and consumed to prevent gastric and duodenal ulcers, in traditional medicine in North and West Africa (37). The *Hericium* fruiting body accommodates copper metalloenzymes (e.g., polyphenol oxidases, SOD, tyrosinase, and laccases), which are multifunctional and have strong antioxidant properties (38).

Proteolysis, the enzymatic hydrolysis of proteins into smaller peptides or amino acids, is vital in many physiological and metabolic mechanisms and pathways in all ecosystems. Basidiomycete mushrooms harbor rich reservoirs of proteases used in vital mechanisms of living organisms, in bioconversion of agro-wastes toward useful substrates, and agricultural and biotechnological processes (39).

In addition to the presence of enzymes, mushroom secondary metabolites can be used in unconventional applications such as processes associated with the development of cancer and as an alternative anti-nematode agent (17, 40, 41).

The large-scale research on proteomes' vanguard technique has been utilized as a sound means to investigate the biophysical properties of the protein fraction of edible mushrooms, namely Basidiomycota phyla, focussing on the molecular interactions involved in developmental milestones (42).

This includes bioactive peptides, released and active after the fractionation of proteins, as a promising strategy with potential or already involved in the synthesis of elements with anti-cancer, anti-inflammatory, anti-hypertensive, anti-microbial, anti-diabetic, antioxidant, anti-biotic properties, and in other responses to environmental changes (43).

Nucleosides and nucleotides take part in the onset and maintenance of energy for anabolism, the synthesis of polymers, and in interspecies interaction between cells, interacting with cell surface protein binding receptors. This enables the transmission of communication and exchanges within the cell and, therefore, the regulation of biological phenomena in the human body through the wide spectrum of purinergic and pyrimidine surface receptors (44).

The distribution of purine nucleobases (adenine and guanine), pyrimidine nucleobases (cytosine, uracil, and thymine), nucleosides (uridine, guanosine, adenosine, and cytidine), and novel monomeric units for nucleic acids (nucleosides and nucleotides) in edible and non-edible mushrooms needs further studies (45).

Redox imbalance generated from by-products of normal cellular metabolism spawns a vast number of modified nucleotides, created by multi-enzymatic cascade reactions and by spontaneous, without energy input, chemical reactions (46).

Elevated levels of blood nucleic acids have been related to several disorders and aging. Possible neurotoxic effects of nucleic acids from foods were investigated and the determination of levels of nucleic acid in several mushrooms showed that it is safe to consume mushrooms as daily food (47). Nevertheless, when studying *Agaricus bisporus* microRNAs (miRNAs), it was considered that they may interfere with important biological processes related to cancer, infection, and neurodegenerative diseases (48).

The few available viricidal and antiviral therapies can be complemented with nutraceuticals from mushrooms epigenetically active. MicroRNAs post-transcriptionally regulate viral and host gene expression by controlling the expression of their target messenger RNAs.

Mushroom species have been identified by cell-free nucleic acids also used as molecular signatures for prospective cancer biomarkers (49). Recently, nucleic acid-based biosensors gained importance due to their broad usefulness in monitoring parameters, which is very important in the fields of clinical diagnosis, drug development, and the food industry, among others (50).

**Figure 2:**
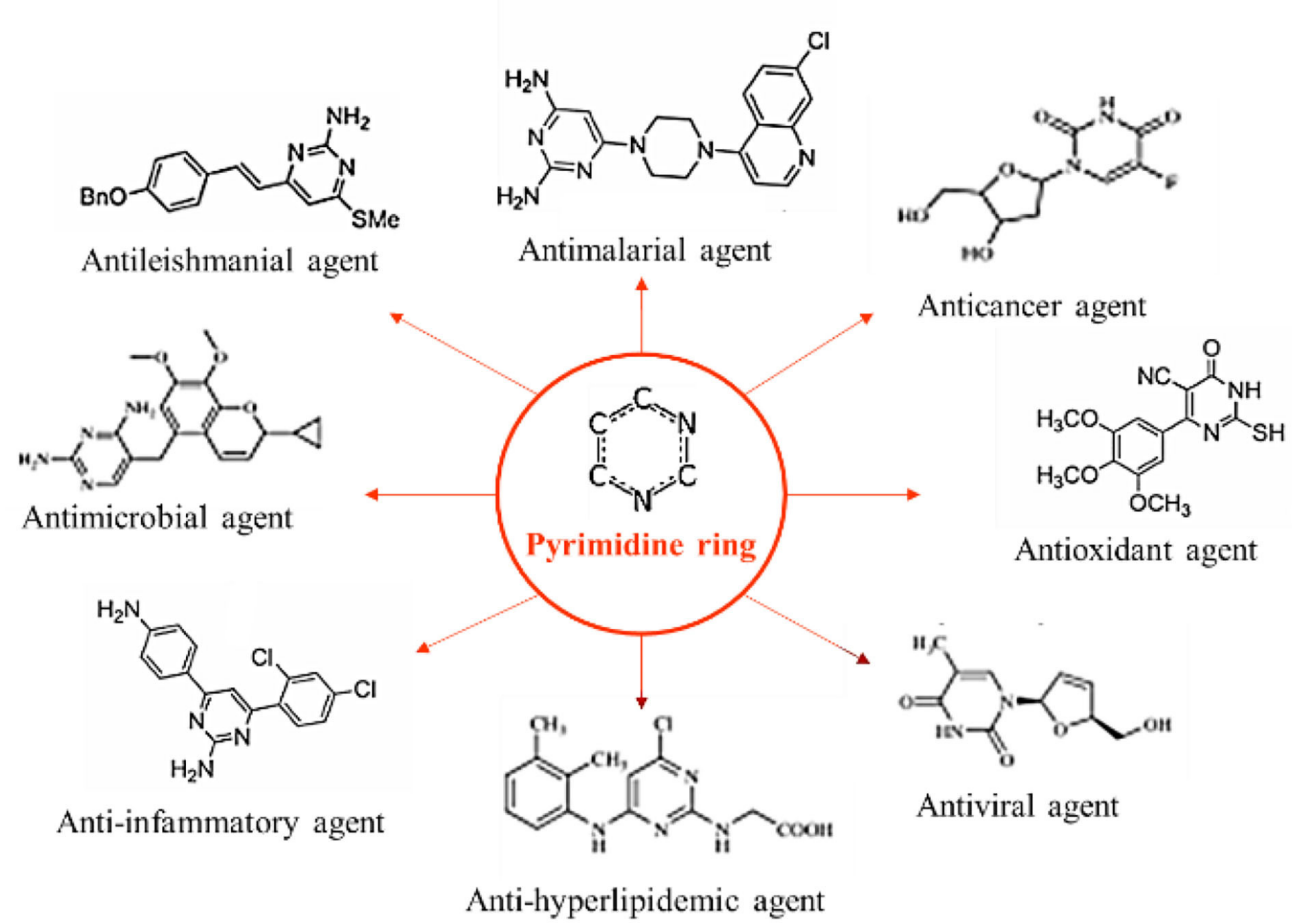
Wide range of potential functions of pyrimidine derivatives in several health conditions.

Various therapeutic platforms delivering oligonucleotide drugs have been established and licensed, although limited by the challenges of safety and efficient consignment (51). The pyrimidine ring structure (along with purines) serves as the informational monomers of RNA and DNA, features of the gene pool, and has been disclosed to have curative prospects and valuable biomedical claims (Figure 2).

Nucleic acids are present in most foods, and meat, fish, seafood, legumes, and mushrooms contain the highest levels of these compounds varying from 2 to 6 g nucleic acid per 100 g crude protein. Targeting nucleotide (purines and pyrimidines) metabolism may stimulate the immune system and combined with immunotherapeutic treatment may control various malignancies (52).

Dietary purines produce dose-proportional increases in plasma uric acid concentrations and renal excretion, responsible for gout and renal calculi, and may influence the biosynthesis of pyrimidines, although the understanding of the inter-relationships of purine and pyrimidine metabolism requires both a global and targeted study of unprecedented scale, limited by the technology available (53).

*Cordyceps sinensis*, a mushroom that contains cordycepin, a derivative of the nucleoside adenosine and activator of its receptors, stimulates the production of interleukin 10, an anti-inflammatory cytokine (54). It is claimed that cordycepin products could be used as a potential medicinal adenosine receptor agonist, which could play a favorable part in the relief of COVID-19 pneumonia and the protection of the brain (55).

Mushroom secondary metabolites triterpenes, abundant in reishi mushroom (*Ganoderma lucidum*), overpower various pro-inflammation markers such as TNF-α, interleukin-6, nitrogen monoxide free radical, prostaglandin E, nuclear factor kappa B (NF-κB), and cyclooxygenase-2 (COX-2) (56, 57).

*Poria cocos* mushroom, besides their high content in oligosaccharides and glycoproteins, incorporates lanostane triterpenoids, showed no immunotoxicity, and was observed to improve inflammation and treat tumors (58).

Other mushrooms deploy anti-inflammatory effects indirectly, by smothering harmful free radicals and preventing oxidative damage. Chaga mushroom (*Inonotus obliquus*), for instance, has antioxidant activity, protecting cells against oxidative damage *in vitro* (59, 60). Oyster mushroom (*Pleurotus ostreatus*) has an antioxidant effect and showed no cytotoxic activity (61).

The positive health effects of mushroom consumption during inflammation have been demonstrated by inhibiting the production of pro-inflammatory mediators (62). However, there were modest recorded effects of *in vivo* consumption of edible mushrooms on induced inflammatory responses. The result is foreseeable since it would inevitably be detrimental if strongly instigated or hampering immune function after intake of a commonly ingested food (63).

Mushrooms are rich sources of dietary fiber with two main components: soluble fiber and insoluble fiber, richer in stems rather than in caps. The insoluble fraction includes chitin (some 5 g/100 gDM) and oligosaccharides β-glucans, being the most representative. Others, such as heteropolysaccharides (e.g., pectineus substances, hemicellulose, and polyuronides), make overall as much as 10–50% of DM. The interference of these fibers on nutrient bioaccessibility has been the subject of many studies taking into consideration their diversity, types, and quality (64).

Much of the diverse array of bioactive molecules includes active polysaccharide peptides found in the mycelium, while the fruiting body mainly contains oligo- and polysaccharide β-glucans (65). The interest in these bioactive compounds is extended to their potential in the prevention and treatment of COVID-19 (66).

These dietary healthy fibers cannot be metabolized by enzymes encoded in the human genome but can be degraded and fermented by enzymes of some resident species of gut microbiota. Although not all β-glucans are capable to modulate immune functions (67), mushroom β-glucans contain β-1, 3-glucan linkages and occasionally β-1, 6 linkages, a structure which is recognized by specific receptors located on the surface of immune cells and conferring immunomodulating effects (68).

Mushroom non-cellulosic β-glucans act as “biological response modifiers” or “biotherapy,” a type of treatment that uses substances contrived from living organisms to treat disease, enhancing the body's own use of macrophages and T-lymphocytes, rather than directly attacking any tumors (69).

Despite their high-molecular-weight, oligosaccharide β-(1,3)-glucans and similar compounds are absorbed *via* M cells of the intestinal cell wall into the lymph, where their fragments are captured by macrophages, subsequently released and conveyed by other granulocytes, monocytes, and dendritic immune cells, leading to various immune responses and functions (70, 71).

Mushrooms were reported to stimulate cell surface receptor activity, enhancing the activity of NK (natural killer) cells, neutrophils, and macrophages, which are the backbone of the innate immune system, thus responsible for antiviral and anti-tumor responses (72). Mushrooms also have the aptitude to support, enhance, or activate the humoral and cellular adaptive immunity system, much slower than an innate response, after initial risk subjection to an antigen or pathogen (73).

*Coriolus versicolor* extract, containing polysaccharide K (PSK) and polysaccharide peptide (PSP), is able to immunomodulate and induce the production of lymphocytes and cytokines, such as interferons and interleukins, exhibiting antioxidant activities (74) and used in anti-cancer mycotherapy (75).

Neuroinflammation is a specialized immune response that occurs in the central nervous system (CNS), connected to chronic neurodegenerative disorders (e.g., ALS—amyotrophic lateral sclerosis, MS—multiple sclerosis, Parkinson's disease, and particularly Alzheimer's disease) that negatively affect mental and physical functioning and are characterized by synaptic dysfunction and a gradual loss of neurons from specific regions (76).

The mitochondria participate in critical central metabolic energy-yielding pathways with vital functions in cellular senescence and the pathological mechanisms underlying cancer, neurodegenerative, and other diseases. Mitochondrial function emerges at the interface of determining health and disease, controls intestinal epithelial stem cell niche, crucial for tissue homeostasis, self-renewal, and differentiation, which can be affected by diet as nutritional changes can alter mitochondrial morphology, energy metabolism, and dynamics (77).

Mushrooms and cyanobacteria (known as blue-green algae) *Spirulina* contain a unique naturally occurring specific molecule, which humans are unable to synthesize, the amino acid ergothioneine (range between 0.06 and 5.54 mg/g DM), with powerful metabolic properties, and considered the last undiscovered vitamin (78). Ergothioneine from diet accumulates in many other tissues, and there has been a myriad of alternative applications, playing a critical role in human development and health (72, 79).

For many years PSP, pleuran, lentinan, grifolan, krestin-PSK, schizophyllan, and scleroglucan have been well-described bioactive polysaccharides from the mushroom origin, used as biopharmaceuticals with widespread clinical significance (75).

## Mushrooms' omission in dietary guidelines

Globally, protein requirements have been organized under the concept of being either animal- or plant-based disregarding other substantial protein sources such as mycoprotein has been widely neglected in all dietary guidelines (80).

The daily intake of mushrooms worldwide, namely in western countries, has been rather low and considered an extravagant food delicacy; however, their incorporation in diets grants the crucial provision of micronutrients and medicinal bioactive components (81).

Mushrooms have a single and vital nutrient portrait reinforcing the general suggestion of consuming lower energy and sodium, and they are uniquely high in bioavailable and stable vitamin D2. The nutritional impact of adding mushrooms to current dietary intakes has been studied (82).

Fungi products are commercialized as fresh or dried, as biomass dietary supplements, or as extracts. This has an impact on marketing since mushroom extracts are legally considered medicinal nutraceuticals or drugs, while biomass is deemed as foodstuffs or dietary supplements (83).

However, the world general legislation governing dietary supplements rests imprecise because there is no general agreement and they can either be considered as foodstuffs and/or medicinal products as determined by various factors (19, 84, 85).

A harmonious and broad-ranging “Food Guideline” should be eclectic and comprise advice concerning various food categories, food safety elements, provision of meals, volume of (un)refined foodstuffs, processing phases, traditional food items, fermented foods and beverages, salt and sugar maximum levels of intake, sociocultural habits, and even creed and religious faiths (86).

Even considering developed countries, only in 2021, there has been a pioneer recommendation on food guidelines of adding a serving (84 g/day) of mushroom mixtures to USDA Food Patterns, the equivalent of five medium white mushrooms (87).

## Anti-inflammatory activity of mushrooms

Inflammation is an immune reaction that shares the complex biological response to detrimental injury, infection, or disease. It is a physiological protective mechanism and a necessary part of healing (88). Evidence supporting the impact of specific foods on chronic persistent inflammation in the body is limited (89). Inflammation is useful because it is the result of the body fighting the infection; however, it is adverse because it damages a lot of the healthy cells in the process.

Inflammation, the fundamental principle of pathology, indicative key of injury and disease, is a critical component of metabolic syndrome, consisting of a carefully controlled flow stage-managed by immune signaling responses termed pro-inflammatory cytokines (IL-1, IL-6, and TNF-α.) and chemokines (90).

Inflammation can be suppressed by ingestion of some foods; however, diet is not the sole factor, and it is intangible the frequency and the abundance required for this advantage. Though there is promising research on the impact of particular foods, with no attention given to long-term eating habits and an anti-inflammatory lifestyle, there is no anti-inflammatory miracle food and although the diet is important, it is not the single cause.

To escape chronic inflammation, it is important to follow healthy dietary patterns, avoiding pro-inflammation diets based on processed red meat, carbohydrates, fried chips, fizzy drinks, and processed sausages. Although there is epidemiological evidence on the benefit of several phytonutrients and other factors in health, only limited clinical data share this outlook (91).

The vast list on news media of alleged anti-inflammatory foods is sometimes super-misleading and not evidence-based. The inventory includes mushroom metabolites, berries rich in anthocyanins and polyphenols, fatty fish and olive oil sources of omega-3s, broccoli rich in sulforaphane, avocados containing carotenoids, peppers providing quercetin, green tea with epigallocatechin-3-gallate (EGCG), grapes supplying anthocyanins, turmeric providing curcumin, tomatoes rich in lycopene, and dark chocolate assigning flavanols.

Mushroom extracts and bioactive components were proven to display anti-inflammatory activity serving as a clinical tool for the natural, safe, and controlled reduction of inflammation and as alternatives to conventional therapeutics (92).

*Agaricus, Pleurotus, Cordyceps*, and *Coriolus* are the most researched species, having the ability to reduce inflammation, but it is unclear how often and how much is needed to ingest for this benefit. Through diet, inflammation may be reduced or restrained, but it must be noted that there is no anti-inflammatory marvel food, since it is not the sole factor involved and lifestyle is also crucial (93).

**Figure 3:**
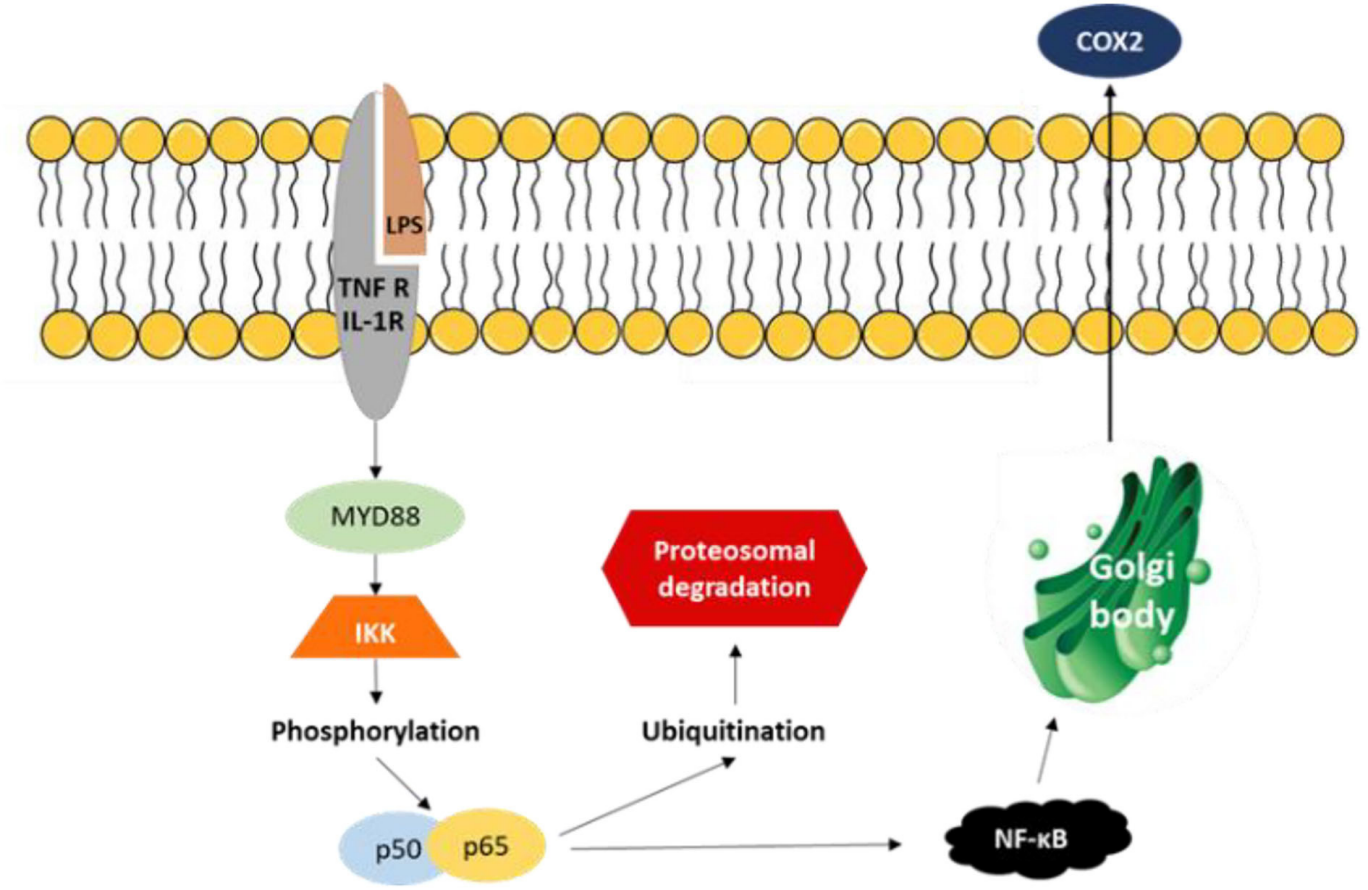
TNF R, tumor necrosis factor receptors; TLR ligands (class of proteins that play a key role in the innate immune system); LPS, lipopolysaccharides; IL-1R, interleukin-1 receptor; MYD88—innate immune signal transduction adaptor—provides instructions for making a protein involved in signaling within immune cells; NF-κB is a major transcription factor, essential for inflammatory responses, that regulates genes responsible for both the innate and adaptive immune response; IKK (classical Kinases responsible for the activation of NF-κB); p50 and p65, subunits of NF-kappa B; COX2, enzyme inhibitor that suppresses inflammatory pathways; proteasome degradation: protein complexes that degrade unneeded or damaged proteins by proteolysis; ubiquitination: a protein is inactivated by attaching a single-chain polypeptide (ubiquitin) to it.

Mushrooms also have an effect on immune function, but that effect is evident only when the immune system is challenged (86). Mushrooms have anti-inflammatory control by suppressing the NF-κB signaling pathways, which control the expression of cytokines, chemokines, adhesion molecules, and cell survival. Bioactive compounds of mushrooms target this major transcription factor highly implicated in both the onset and resolution of acute inflammation and activation of the immune system (Figure 3).

**Figure 4:**
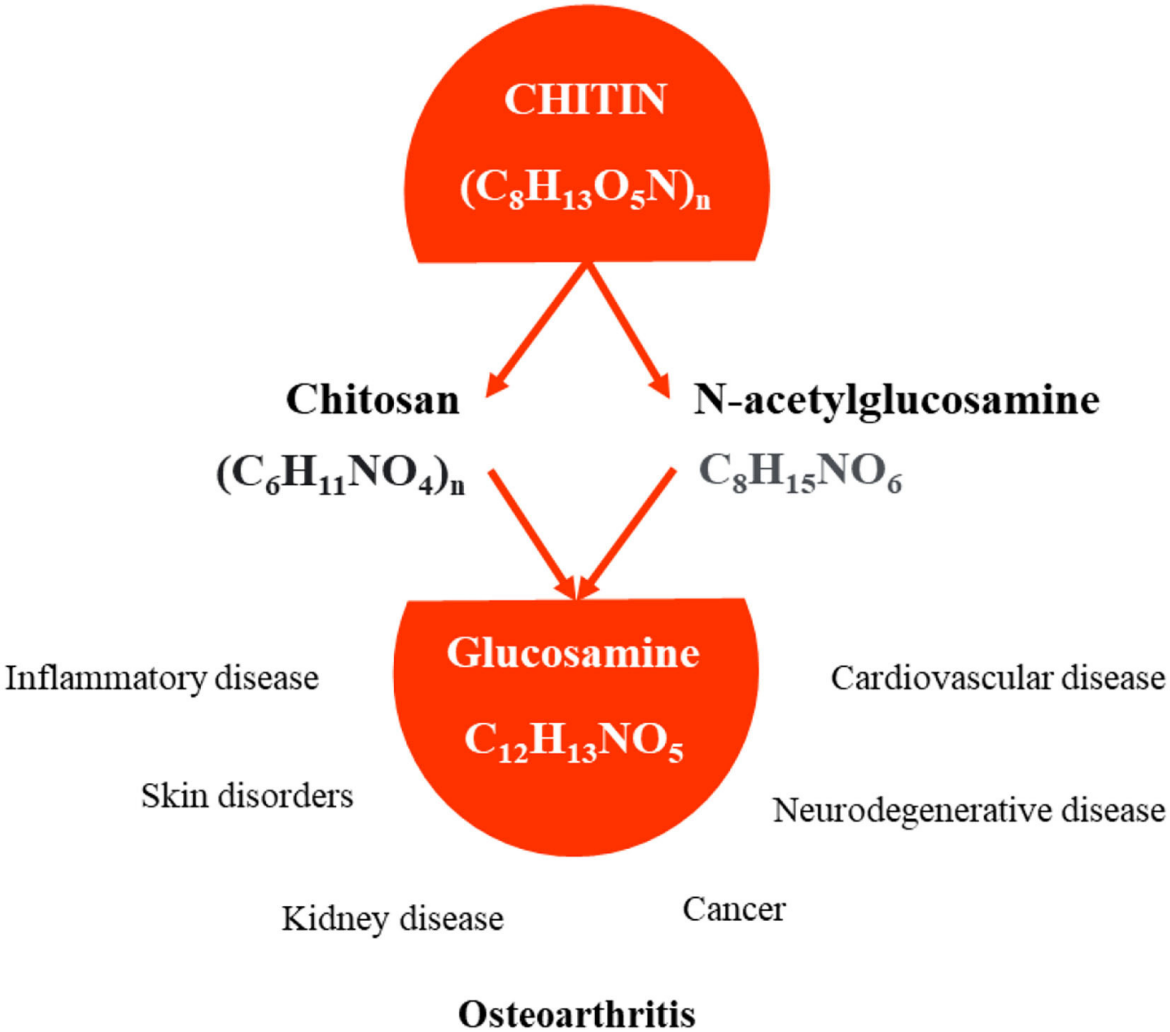
Although the effects of glucosamine are still unclear and controversial, it has been widely used as a dietary supplement for relieving complaints of osteoarthritis.

After plant cellulose, chitin is the most abundant biodegradable polymer in nature. Several mushrooms (e.g., *Pleurotus, Termitomyces, Lactarius*, and *Agaricus*) are rich in chitin (C_8_H_13_O_5_N)_n_, a long-chain polymer of N-acetylglucosamine, and the main component of the cell walls of fungi, insects, shrimp, and crustaceans shells (94). Chitin can be decomposed into glucosamine, which is involved in the creation of molecules that protect joints from inflammation and shields from other impacts on health (Figure 4).

Other mushrooms act directly on inflammation, and *Agaricus blaz*ei, *Hericium erinaceus*, and *Grifola frondosa* have been shown to exert this activity even in severe lung inflammation that often follows COVID-19 infection (95).

*Cordyceps* spp, entomopathogenic fungi, enclosing more than 700 species of the same genus, originated from the Tibet region, is widely used in Chinese medicine, and *C. sinensis* and *C. militaris* being the most frequently used in a wide range of biotherapeutic activities (54).

This mushroom is plentiful and assorted in highlands in humid temperate and tropical forests of the high Tibetan Plateau and contains a low-molecular-weight nucleoside compound, cordycepin, which stimulates the production of interleukin 10, an anti-inflammatory cytokine (96).

*Poria cocos* mushrooms incorporate hydrocarbon terpenes, described to improve inflammation and treat cancers (97). Other macrofungi deploy an indirect anti-inflammatory effect by curbing harmful free radicals and hindering oxidation. Chaga mushrooms (*Inonotus obliquus*) bestow antioxidant activity, shielding cells against redox imbalance (98). Oyster mushrooms (*Pleurotus ostreatus*) also show an antioxidant effect, preventing damage to DNA and cell membranes (99).

It is important to understand the difference in the chemical nature of extracted β-glucan from various sources. Cereals contain β-glucans at different levels, some 2.5 % (rye and wheat) up to 4.5% (oats and barley), but these are not capable of controlling immune functions, as they are usually processed prior to intake (100).

They are nevertheless considered beneficial for lowering the postprandial glucose response and the improvement of blood cholesterol levels (101). However, mushroom β-glucans, consisting mostly of 1,3-β linked glycopyranosyl residues with small numbers of (1,6)-β-linked side chains, operate as bioactive molecules with immunomodulating properties (102).

**Figure 5:**
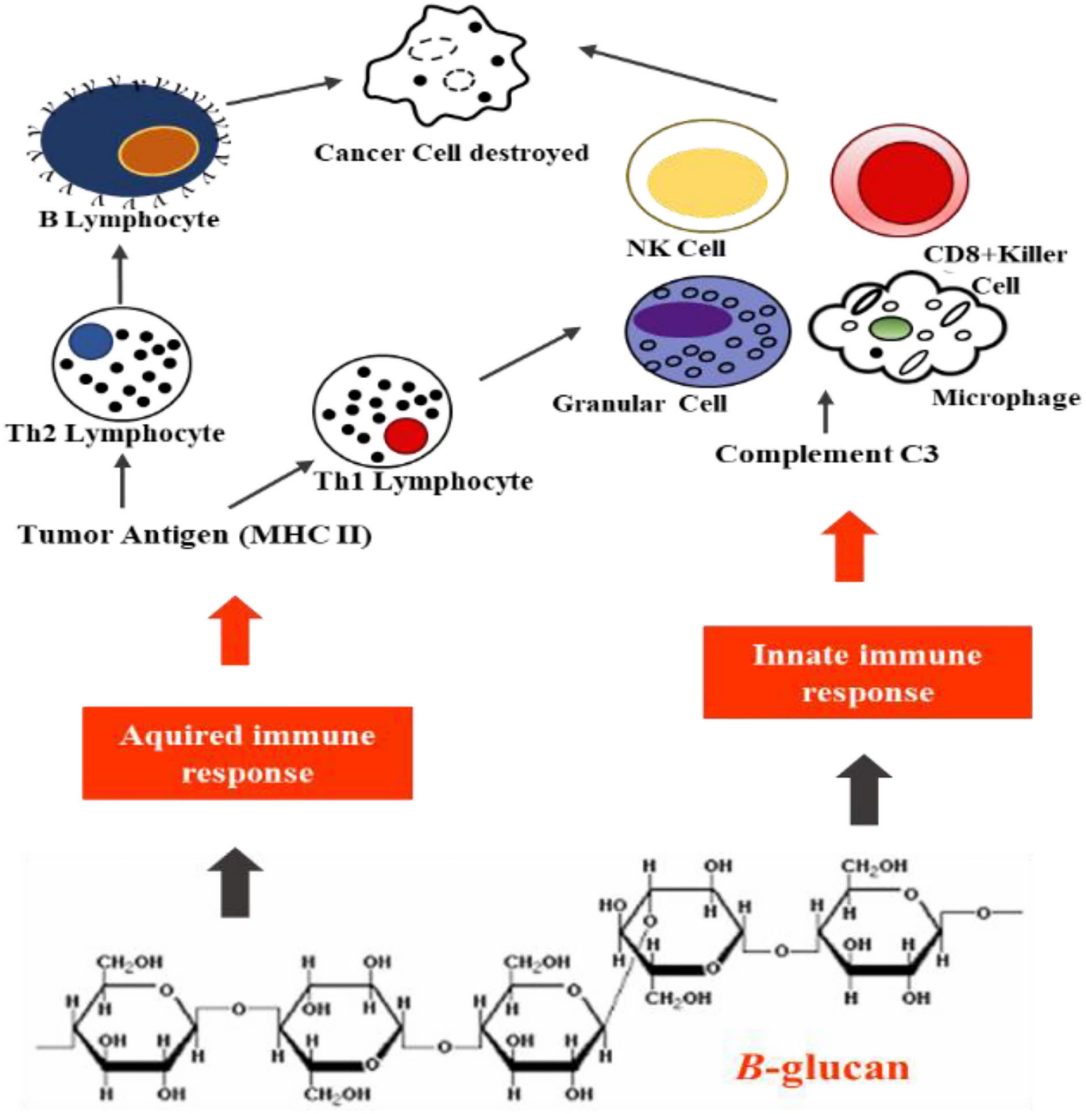
System of antineoplastic activity of bioactive β-glucan. The complexity and multi-step cancer cell recognition by the immune system, and the possible therapeutic applications of tumor-specific major histocompatibility complex (MHC-II). Normally, tumor cells do not express MHC-II genes.

These BRD-biological response modifiers (1,3)-β-glucans interlink with the brush border enterocyte villi cells and are absorbed into the lymph capillaries (103). Here, they enroll the immune sentinel cells, neutrophils, and macrophages, and activate the production of cytokines crucial in controlling and stimulating immune function (Figure 5).

Neuroinflammation is a specialized immune response that occurs in the central nervous system, and it is linked to chronic neurodegenerative disorders (e.g., amyotrophic lateral sclerosis-ALS, multiple sclerosis, Huntington's disease, Parkinson's disease, and particularly Alzheimer's), negatively affecting mental and physical functioning being characterized by synaptic dysfunction and a gradual loss of neurons from specific regions (104).

**Figure 6:**
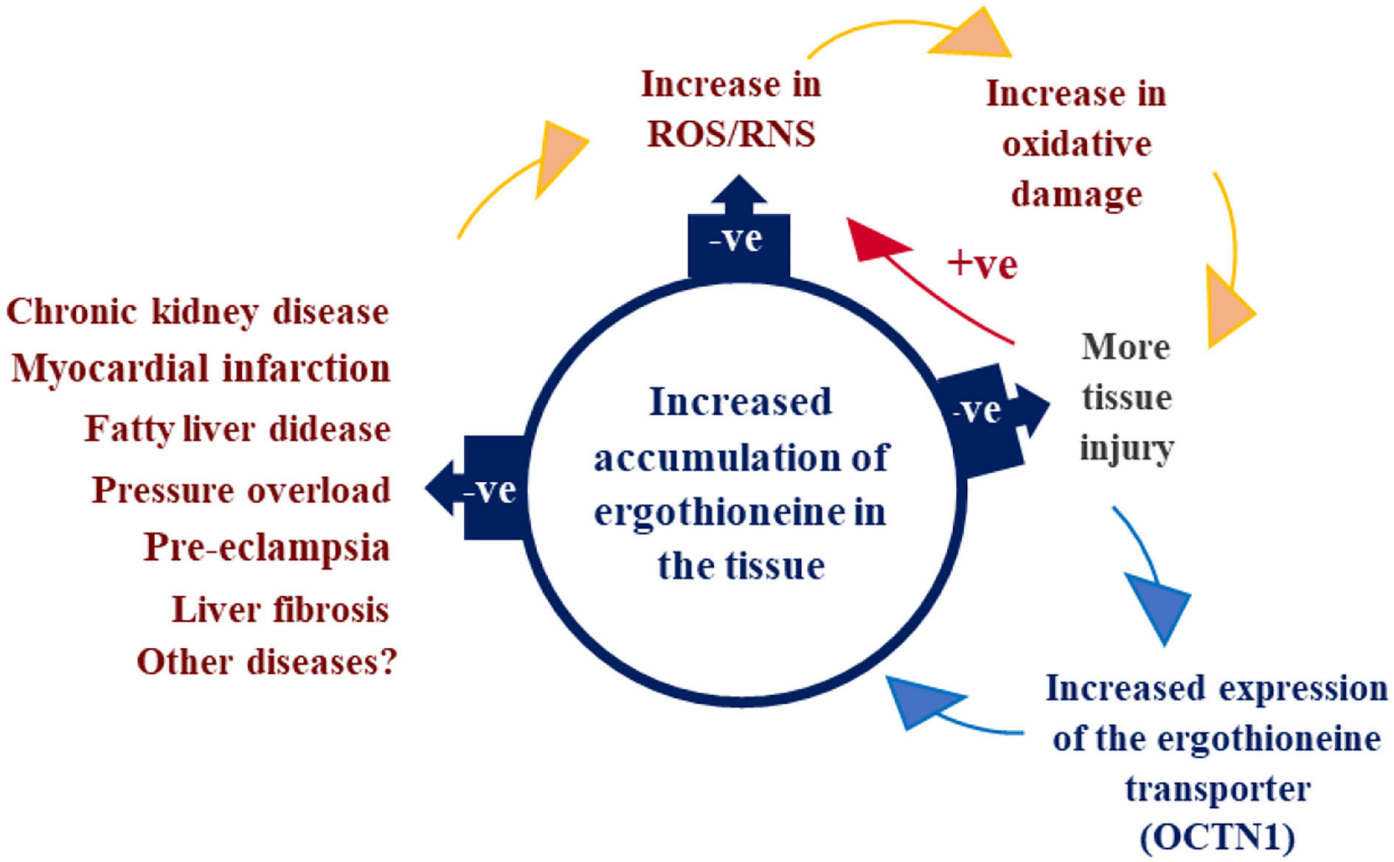
Ergothioneine, a potent intra-mitochondrial antioxidant. A diet-derived antioxidant with therapeutic potential, approved by EFSA and FDA, referred to as a “longevity vitamin”.

Mushrooms integrate and are excellent suppliers of ergothioneine, compound humans cannot produce, a distinctive antioxidant, cytoprotective, and anti-inflammatory stem from food histidine. The unique sulfur-containing antioxidant on a deliberate cytoprotective mechanism can accumulate to high levels in red blood cells and many other tissues, functioning both as a therapeutic and possibly as a preventative agent for several diseases (Figure 6).

## Antiviral properties of mushrooms

New viruses surface all the time, in a process called zoonotic spill over, jumping from wildlife and animals to humans, purely by transmutation of an ongoing native virus, and can be major hazards to public health. A virus is a disease-producing pathogen metabolically dormant and made up of a core of genetic material, either DNA or RNA, and an external lipoprotein shell, unable to replicate unless inside the host cell proteins and mechanisms.

The viral replication cycle differs by the viral type, and according to the host species which it infects, yielding new viral genomes and proteins. It involves attachment, penetration, uncoating, replication, assembly, maturation, and release steps (105).

Mushroom biomass and extracts downgrade viral disease by primarily addressing viral attachment and entrance to the cell, affecting genome reproduction, reducing the receptor binding of viral envelope glycoproteins by mushroom polysaccharides and triterpenes, and by influencing immune modulation (106).

Numerous previous studies have demonstrated mushrooms as exhibitors of potential antiviral efficacy, including human exposure to the SARS-CoV-2 virus (24, 107).

**Figure 7:**
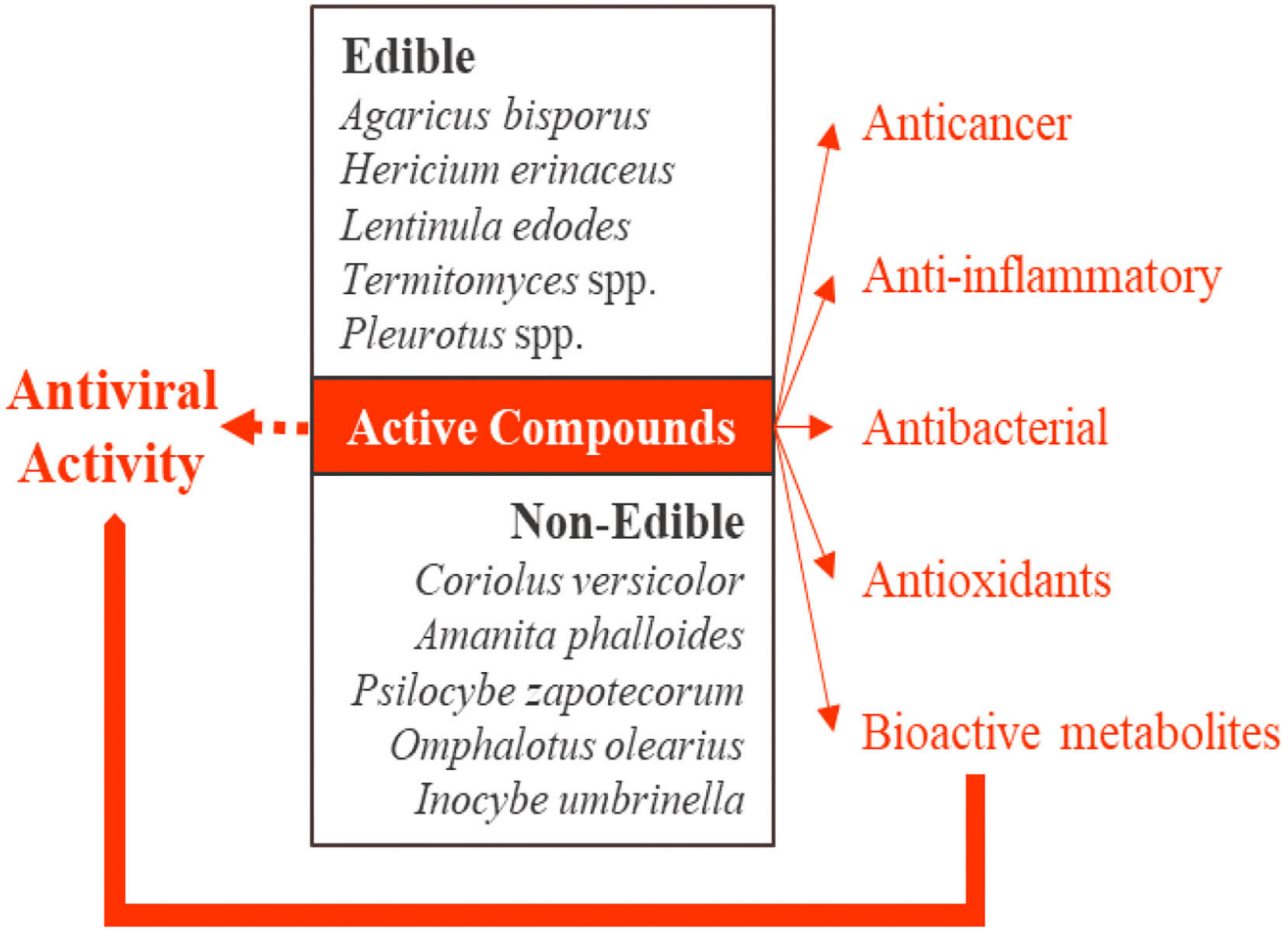
With several 100 bioactive compounds, mushrooms demonstrate antiviral outcomes and are used as dietary supplements, functional food, or medicinal products.

The natural bioactive compounds from mushrooms and dietary health supplements are responsible for the safeguard and therapy of viral infections (108) mainly through the improvement of the activation or suppression of the immune system, where immune responses are induced, amplified, attenuated, or prevented (Figure 7).

The biological active compounds function chiefly by obstructing the entrance of the virus into the cell, by causing the lysis of infected cells by triggering the boosting of NK, CD8+, and T cells, by anti-neuraminidase increased inhibitory activity, and by promotion of cell-mediated immune response (109).

Several clinical studies, specifically for fighting viruses with mushroom products, have enhanced the role of *Cordyceps* fighting the flu virus by boosting the body NK cell activity and other virus-killing cytokines. Herpes simplex and hepatitis C virus were destroyed using *Ganoderma* (reishi). Similar data were obtained with *Grifola* (maitake) and *Lentinula* (shiitake).

### Human immunodeficiency virus (HIV)

The pathogenesis of this disease is considered of multifactorial nature and occurs in communities where malnutrition is endemic and WHO has long established the nutrient requirements for people living with HIV/AIDS (110).

Extensive comprehensive protection for people living with HIV/AIDS requires nutritional care mediation, bioactive molecules of mushrooms may play crucial assistance to patients, and the nutritional guidelines previously established for diet management need updating, especially in limited-resource settings (105).

The evaluation of the complex interplay between HIV/AIDS and under-nutrition and the degree of severity is crucial to forecast the evolution of the disease and the probability of morbidity and death toll, namely in children (111).

Mushrooms may play a decisive role since β-glucans activate the complement system, increase CD4 cell production, boost NK cell production, and stimulate the immune system macrophages (112). Even when infected with HIV, the macrophages fight effectively, reducing HIV replication and mediators of chronic inflammation.

Many studies reveal that several triterpenes from *Ganoderma lucidum* act as antiviral agents against HIV type 1 protease (113) or by boosting the immune system indirectly blocking its multiplication (114).

Direct antiviral consequences involve blockage of viral enzymes and synthesis of viral nucleic acids and adsorption or uptake of viruses. Indirect antiviral effects are completed by vitalizing the immune response or developing biochemical factors, such as pH increase, which interdicts viral replication (115). Mushroom components, namely β-glucans, flavonoids, glycoproteins, melanin pigments, triterpenoids, and nucleosides, have all shown powerful antiviral activity (116).

Several studies revealed that *Ganoderma lucidum, Pleurotus eryngii*, and *Lentinus tigrinus* can yield laccases, cellulase, xylanases, and ligninolytic peroxidases, enzymes that can provide HIV-1 reverse transcriptase inhibitory activity against HIV (117).

The HIV-1 has a lymphotropic nature, meaning that they infect human T cells, causing relentless disruption of the lymphatic system and attached structures like the nodes, spleen, and thymus, rising numbers of dendritic cells, and antigen functions.

HIV-1 infection is currently manageable but resistance to anti-retroviral (ARV) agents is emerging, and many people infected with HIV have serious adverse reactions. Antiviral drugs do not cure HIV infection but suppress viral replication. Antiviral bioactive compounds from mushrooms and other herbal remedies are potent bio remedies, namely in the pre-exposure prevention phase, acting directly on the metabolic pathways of the human host, and regulating the interactions between viral RNAs and host cell proteins (118).

### Herpes Simplex Virus (HSV)

The Herpes Simplex Virus (HSV-1) is developed in humans for millennia in a sustained, active, and perpetual shuffle where the pathogen is present at a high incidence, affecting globally half of the human population, some 3.8 billion people (119).

As over 130 known herpes viruses have been disclosed, eight infect humans and two strains occur in most β-amyloid plaques of Alzheimer's disease (AD). Some two-thirds of their proteins are indistinguishable, hinting that this frequent virus is a potential risk factor for AD, emphasizing that a particular viral species may bluntly constitute a risk of developing AD (120, 121).

The neurotropic virus either persists in an inert attitude, intercalated with reactivation events, or even engenders serious acute nervous syndromes, based on neuroinflammation and secondary effects, able to induce unsafe neural diseases (122).

Once inside the cell, HSVs dramatically modify human metabolism, inducing antiviral immune responses and assigning cell apoptosis in non-immune cells, immune cells, and T cells, while viral reproduction occurs in somatic cells prior to outbreaking into nerve centers, producing a symptomless infection (123).

The anti-herpetic activity of fruit bodies, cultivated mycelia, as supplements or teas and infusions, from *Coriolus versicolor* and several other mushrooms (e.g., *Inonotus, Pleurotus, Fomes, Auriporia*, and *Polyporus*) has been demonstrated for many years, including the antiviral activity against herpes simplex virus type 2 (HSV-2) (124, 125). As with many supplements and medications, the use of these products as therapeutics may also interact with other pharmaceuticals and carry some risks.

### Influenza viruses

Global ecological fluctuations in climate and land may be responsible for zoonotic spill over transmission where a virus circulating quietly may jump from wildlife to humans, causing a disease emergence (126). This is serious in view of the great degree of transmissibility and the ability of these viruses to generate successful escape mutations.

Fresh mushrooms or dietary supplements are successful in intercepting transmission and handling several viruses such as viral rhinitis and the influenza respiratory viruses. *Grifola frondosa, Ganoderma lucidum*, and *Inonotus obliquus* were proven to be effective against the flu-causing influenza viruses (127).

Although a mineral deficiency is rare, some mushrooms are a rich source of specific minerals (e.g., selenium, magnesium, and zinc), which may play a direct or indirect role in their anti-influenza properties, performing a crucial role in boosting the immune system and preventing the viral infections, and preserving the homeostasis process in the human body (128). Dried mushrooms might be a source of mineral components indispensable for human health (129).

### Human papillomaviruses (HPVs)

The administration of *Coriolus versicolor* biomass dietary supplement in women for a period of 1 year showed considerable success in the relapse of the cervical abnormal condition in the low-grade squamous intraepithelial lesion (LSIL) or in the removal of the high-risk HPV that can cause cancer (130).

Also, the efficacy of a *Coriolus versicolor*-based vaginal gel in women with human papillomavirus-dependent cervical lesions was positively evaluated in repairing mucosa lesions with low-grade Pap smear alterations (131).

This was replicated with a proprietary compound, active hexose correlated compound (AHCC), a fermented extract of *Lentinula edodes* mycelia, following administration for at least 6 months with a 60% successful elimination of human papillomavirus (HPV) infections in women with positive PAP (132).

Mushroom biomass forms, like other natural regimens, appear a safe treatment and are usually administered as a dietary supplement in combination with other treatments and safely boosting radiation and chemotherapeutic efficacy, as well as surgery, with a significant impact on cytotoxic activity of natural killer (NK) and T cells which play important roles in host immune defense.

### The novel coronavirus (SARS-CoV-2)

The research has been intensive during the past 2 years, and there is a vast abundance of publications on COVID-19, while currently only one medication has been approved for this disease. The curious fact about the SARS-CoV-2 virus is the disparity of symptoms, ranging from no tenable manifestations to extensive signs of severe disease, namely pneumonia with fever, dry cough, and dyspnoea, spreading the virus and transmitting the disease.

Data on the role of mushrooms in this pandemic are still scarce. Indeed, clinical trials are presently underway, sanctioned by the FDA, with two polypore mushrooms, *Fomitopsis officinalis* and *Coriolus versicolor* known to strongly induce an array of differential cytokine responses (133).

The immunomodulation influential properties of *Cordyceps sinensis* (only grow in nature) and *Cordyceps militaris* were evaluated and considered fine for the prevention and treatment of COVID-19, as they are traditionally used mushrooms to improve lung functions. As severe disease indicates inflammatory response and cytokine storm plays a crucial role in the disease's pathophysiology, it is imperative to reduce the pro-inflammatory cytokines, avoid chronic interstitial pneumonitis, normalize tolerance to low levels of oxygen in the blood, and obstruct viral enzymes (134).

Several edible mushroom species including shiitake (*Lentinus edodes*), Chaga (*Inonotus obliquus*), and maitake (*Grifola* frondosa) are established as natural antivirals but not yet used for treatment against SARS-CoV-2, a present great threat to public health and global economies (66).

As the possibilities of natural substances as effective treatments against COVID-19 seemed promising, the popular mushrooms, white button (*Agaricus blazei*), reishi (*Ganoderma lucidum*), lion's mane (*Hericium erinaceus*), and maitake (*Grifola frondosa*) were tested in Norway and regarded to have prophylactic or healing outcomes upon the critical respiratory diseases frequently occurring in combination with COVID-19 infection (95).

*Ganoderma lucidum* consumption showed in Iraq to have a prominent role in health assessment through the diagnostic and prognostic value of the hematological and immunological parameters in 150 patients with COVID-19, reducing the number of infections and assisting in the treatment of the pandemic (135).

**Figure 8:**
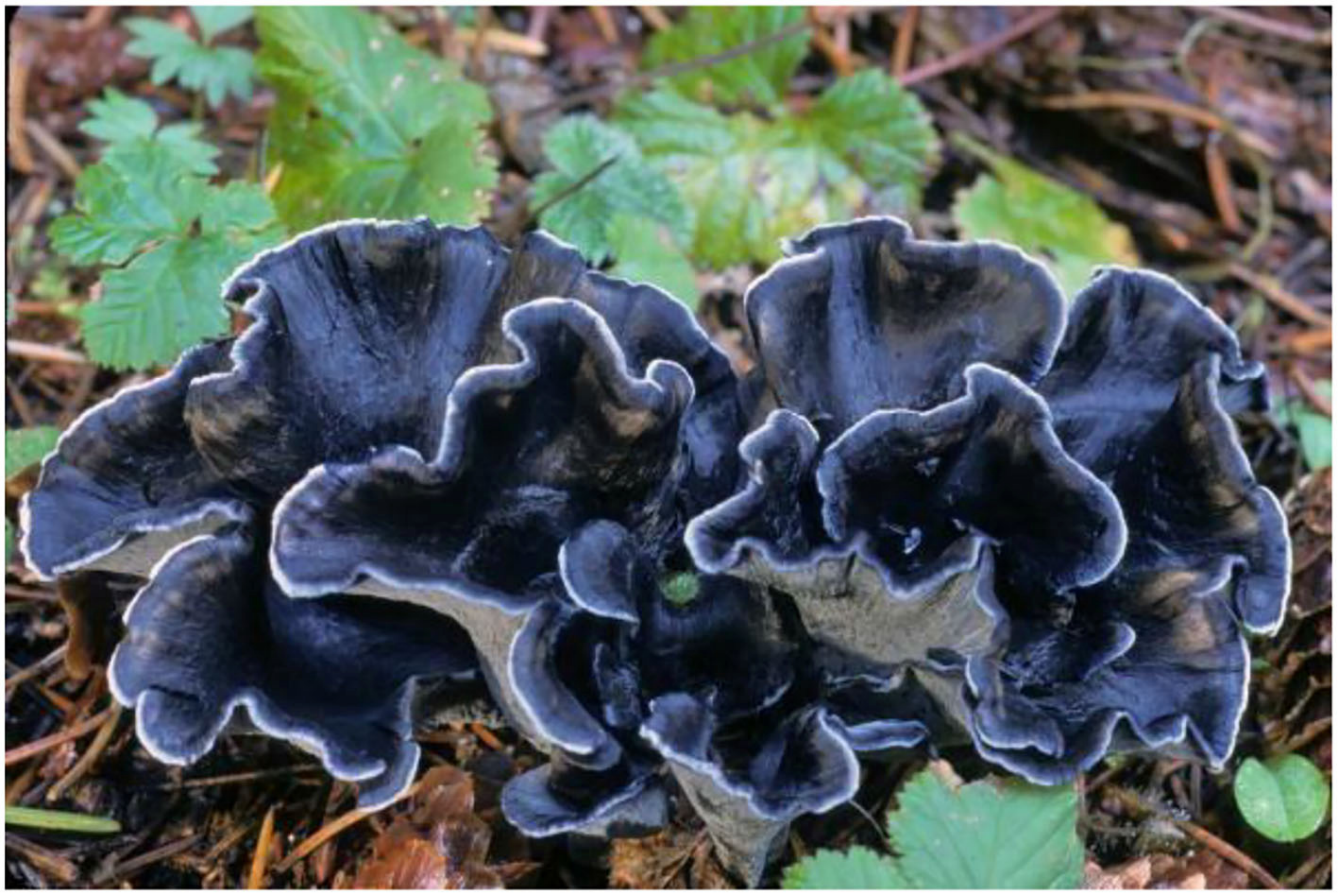
Blue chanterelle mushroom *Polyozellus multiplex* from the family *Thelephoraceae*.

Encouraging impact counteracting the main protease (Mpro) of SARS-CoV-2 from edible mushrooms has recently been disclosed in January 2022 in India and concluded that edible mushroom blue chanterelle *Polyozellus multiplex* (Figure 8) has the aptitude for controlling SARS-CoV-2 infection and determined the elements that could be further studied as medicinal barriers against SARS-CoV-2 (136).

Other unpublished investigation strongly supports that the natural bioactive compounds from edible mushrooms and marine fungi have promising therapeutic potential which can be further exploited for the rapid development of nutraceutical against different virus including the SARS-CoV-2 virus (137).

New data indicate that polysaccharides from the mushroom Chaga (*Inonotus obliquus*) are potent natural resources for antiviral therapy as an adjuvant to anti-SARS-CoV-2 vaccination and medicaments. Indeed, the envelope S1 spike glycoprotein, the main antigen component, of coronavirus assists the entrance of the virus into the host cell. Some Chaga components specifically interact and attach with the terminal domains of the SARS-CoV-2 S-proteins, regulating its receptors and inhibiting virus entry into the host shelter cell (138).

From the aforementioned, even mushrooms having high safety margins cannot be deduced or recommended mushroom use as functional foods, for SARS-CoV-2 infection, since the focus is to mobilize the non-specific immunity to precipitate prompt antiviral immune natural resistance (139).

Moreover, there have not been adequate long-term risk/benefit assessments of specific mushrooms commonly suggested for pulmonary illness within the frame of the COVID-19 pandemic as an adjuvant treatment.

## Anti-tumor activity of mushrooms

Most cancers or tumors are chronic multifactorial, involving single or combined genetic, environmental, lifestyle, medicinal, and other articles, including a third being clinical studies, revealed the significant impact of administration of *Ganoderma lucidum* and *Coriolus versicolor* mushrooms on the elimination of detrimental health consequences, improving lifestyle quality in oncological patients (140).

Lectins, non-immunoglobulin sugar-binding, and cell-agglutinating proteins are universally distributed in viruses, microorganisms, algae, animals, and plants and are reported as modulators *in vivo* and *in vitro*. These molecules also play a role in the induction of mitosis and immune responses, contributing to the resolution of infections and inflammations (141).

**Figure 9:**
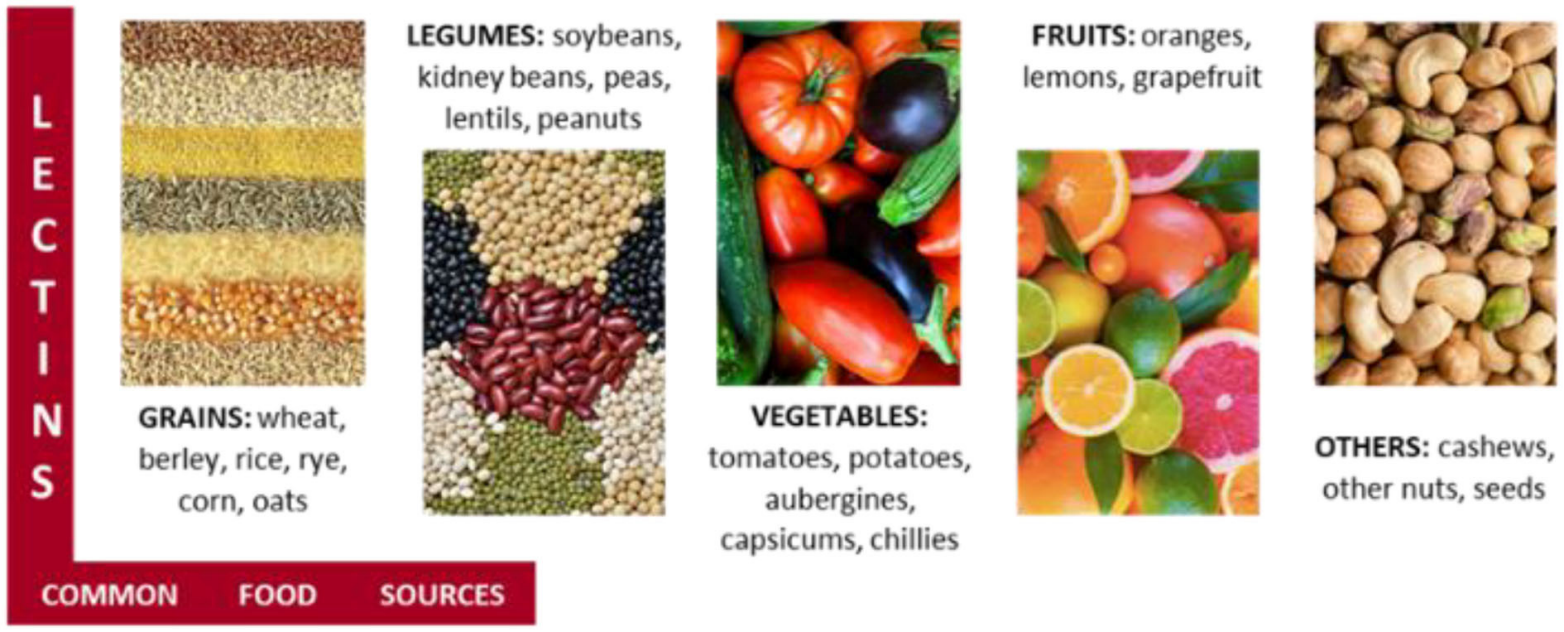
Food sources of anti-nutrient lectins may negatively impact gut permeability and alter the population of good microbiota. Mushrooms' lectins, however, show potential for treating several ailments.

There are several types of lectins (Figure 9) that perform different functions. For example, plants use lectins to defend themselves from harmful microorganisms and insects. Some lectins are good and harmless, others are not, depending on food processing. Lectins are used not only as molecular glue for protein immobilization but also serve as a recognition element of biosensors and have high potential in the diagnosis and therapeutic of diseases (142).

More than 100 mushroom lectins have been identified (143) which exhibit a diversity of chemical characteristics possessing immune modulating and/or direct cytotoxic activity toward tumor cell lines (144).

The lectins found in some mushrooms have higher specificity for N-acetyl galactosamine and are known to possess antiviral and immune-stimulating properties to fight certain illnesses (145). Numerous lectins from mushrooms have shown potent inhibitory activity toward cancer cells through fungal extracellular ribonucleases acting as immunotoxins and are characterized by exerting their cellular cytotoxicity by inactivating ribosomes, leading to protein biosynthesis inhibition and death of neoplastic cells (146, 147).

The anti-tumor activity of mushroom compounds is not by way of direct lysis of the neoplastic cell but by means of mobilization and activation of the body's first line of defense, the non-specific innate immune system born with the host. The operating principle is related to the presence of toll-like receptors agonists that identify mushroom-specific macromolecular polysaccharides as patterns associated with pathogens (148).

As a result, in a cascade of reactions, activated macrophages generate signaling molecules, chemokines, and cytokines (TNF, IL-1β, IL-6, IL-12, and IL-23), which control the balance between immune tolerance and immunogenicity, and onset the processes disputing infections when detecting intruder and neoplastic cells (149, 150).

Fungal cell walls contain chitin, α-glucans, and namely β-glucans polysaccharides with diverse structures and functions. Mushrooms are characterized by the structure of β-1,3-glucans with short β-1,6-side chains which are adapted and recognized by specific receptors. Selected β-glucans from mushrooms (e.g., krestin, grifolan, lentinan, schizophyllan, and pleuran) showed immunomodulatory activity by binding to particular sensory receptors, suggesting a role even in the prevention and treatment of COVID-19 (66, 151).

Anti-tumor activity has been demonstrated from several mushroom components including the macromolecular polysaccharides and the highly oxygenated terpenoids, carotenoids, and steroids. The latter are secondary metabolites from mushroom metabolism, which can regulate cell death through multiple pathways including apoptosis, causing tumor cells to self-destruct, a key part of the immune response (152).

Cancer, globally the leading cause of death, has been the subject of many prophylaxes. Prevention with mushroom products has been used for many years in Asia. Natural components, polysaccharide extracts, from *Hericium erinaceus* are active against hepatocellular carcinoma (153), in gastrointestinal cancers and other types (154).

On an extensive study with almost 100 different mushroom species, it was proven the reduction of the viability of various cancers, including breast tumors (155). High intake of dietary mushrooms, the common *Agaricus bisporus* and *Lentinula edodes*, reduced the risk of breast carcinoma in pre- and postmenopausal women (156).

Oncological patients administered maitake mushroom (*Grifola frondosa*) showed significant control of cancerous growth (157). This was considered due to increased release from the spleen, organ of the lymphatic system, of pro-inflammatory cytokines tumor necrosis factor (TNF)-α and interferon (IFN)-γ, which synergistically enhance NK cell cytotoxicity (158).

**Figure 10:**
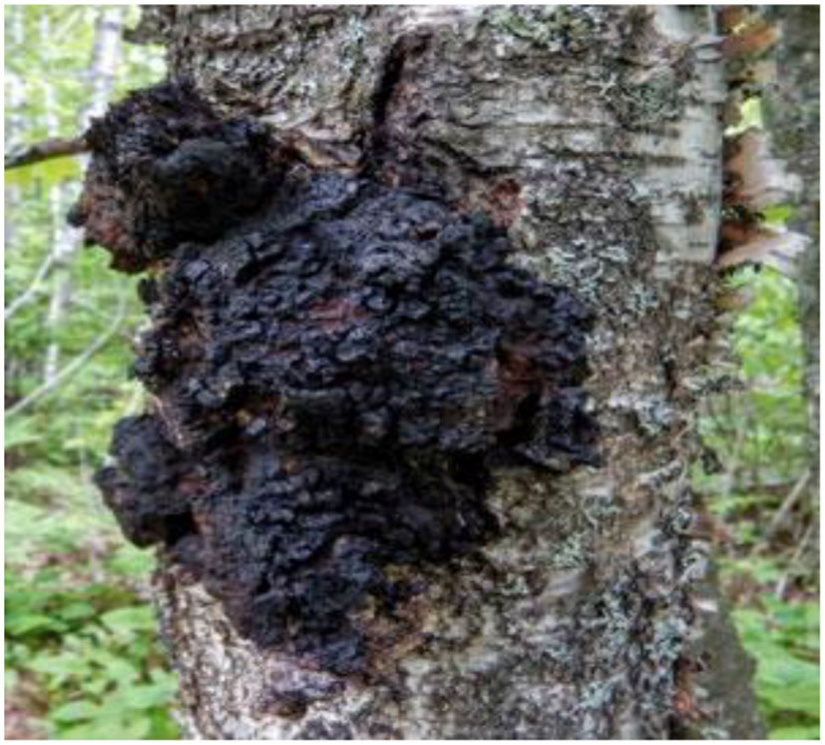
Chaga mushroom (*Inonotus obliquus*) is a parasitic fungus from the family Hymenochaetaceae with potential in oncology.

Chaga mushroom (*Inonotus obliquus*), common in cold climates, is used as tea or extracts from a mixture of wood from the substrate tree and the mycelium of the invasive fungus (Figure 10). It has been evaluated for its potential on most ailments including cancer, demonstrating its antioxidant and immunostimulatory effects both *in vitro* and *in vivo* (159).

Chaga mushroom, used traditionally for beauty and longevity, is a traditional edible mushroom with proven therapeutic value and contains biologically active substances like long-chain homopolysaccharide β-glucan, galactomannan, and the unique terpenoid betulinic acid. However, very few or no clinical trials have assessed Chagas safety or efficacy for disease prevention or treatment of cancer, cardiovascular disease, or diabetes (160).

## Prebiotic activity of mushrooms

Prebiotics, non-digestible food ingredients, act as nourishment for probiotics (e.g., good live microorganisms such as bacteria and yeasts). Prebiotics [e.g., β-glucan, fructo-oligosaccharides (FOS), galacto-oligosaccharides (GOS), trans-galacto-oligosaccharides (TOS), and inulin, a type of oligosaccharide] manifest some direct health benefits such as oral anti-hyperglycaemic and improvement of the gastrointestinal function.

Although they have been medically authenticated and certified by food and health officials, the exact mechanisms behind the prebiotic–gut microbe–host interactions are still under investigation. Edible fungi interact with many beneficial gut microbiota and their non-digestible fiber serves as the substrate for microbial fermentation. Endogenous β-glucans show better prebiotic properties on the modulation of gut microbiota than exogenous β-glucans (148, 161).

β-glucan-producing probiotics may have the prebiotic potential to enhance the growth of other beneficial microbiota in the bowel. In the last decade, the number of patents and scientific articles on biotechnological, nutritional, and therapeutic uses regarding the genus *Pleurotus* has exponentially increased. *Pleurotus ostreatus* and *Pleurotus eryngii* have demonstrated potential catalytic aftermath on the proliferation of probiotic bacteria (162).

The inclusion of mushroom *Coriolus versicolor* in the human diet beneficially affected gut physiological processes and behavioral actions performance and, when the substrate was fermented, influenced modifications in the gut microbiota profile (163).

We have advanced a possible new concept where an ultra-trace metalloid element (e.g., germanium) is said to play an eventual prebiotic complementary role in the mode of action of mushrooms (164). Garlic, ginseng, ginger, and above all mushrooms have significant levels of germanium with significant potential for treating various human illnesses and promoting lifestyle.

## Diabetes

Diabetes mellitus is a non-communicable global disease and a major cause of morbidity and mortality. Increased and chronic inflammation in the body may originate insulin resistance and alter its action which can lead to a cluster of several metabolic conditions, including diabetes and cardiovascular diseases. Mushrooms are rich in soluble and insoluble fiber, which is known to maintain and reduce the body's blood sugar level being effective in controlling glycaemic levels (165).

Prolonged hyperglycaemia, combined with insulin resistance, dyslipidaemia, hypertension, and chronic inflammation, promotes an increase in oxidative stress and vascular damage. This may cause capillary vascular diseases (retinopathy, nephropathy, and neuropathy) or major vascular diseases (coronary heart disease, stroke, or peripheral artery disease), related to type 2 diabetes (166).

Mushrooms and other varieties of fiber-containing foods provide the fiber responsible for decreasing the glycaemic index of diets (167). An adequate intake value of dietary fiber consumption from all diets is some 25–38 g/day (14 g/1,000 kcal/day) (168).

The mechanism of action may be achieved through a reduction in both postprandial plasma glucose and insulin concentrations, but also an increase of magnesium which plays a key role in regulating insulin action, insulin-mediated-glucose-uptake, and vascular tone. Magnesium electrolyte is a co-factor for enzymes regulating insulin and glucose metabolism, such as tyrosine kinase, and occurs in high levels (9%) in raw mushrooms (129).

## Neuroprotective aptitude of mushrooms

Worldwide, there is a preponderance of neurological morbidity and disorders (e.g., mental health, stroke, epilepsy, and dementia), which have been intensifying and are among the main causes of incapacity and even death.

Mitochondria are cytoplasmic organelle authentic cell power plants where energy is produced, and when mutation dysfunctions they are responsible for oxidative stress and the development of neurodegenerative disorders and aging (169). The human body has several strategies to minimize radically induced damages and to control the imbalance between the production of free radicals and the antioxidant system, by either yielding endogenous antioxidant enzymes (e.g., SOD, CAT, and GSH-Px) or through foodstuffs or dietary supplements (170).

Progressive neurodegeneration has a complex etiology and multifactorial pathogenesis is age-related and may derive from disruptions of cellular proteostasis and multiple interactions between circulation and the brain. The bidirectional communication between the brain and gut microbiota may suffer dysregulation, and there is vast evidence that the imbalance of various microbial-derived metabolites and neuroactive compounds may initiate various neurodevelopmental and neurodegenerative diseases (159, 171).

Neuroinflammation is the process whereby the brain's innate immune system is triggered following an inflammatory challenge caused by a virus infection, injury, or toxin exposure, stimulating the host immune system, with various physiological, biochemical, and behavioral consequences. It may result in neuronal disorder and degeneration with irreversible loss of axons and neurons (172).

Mushroom incorporation in diets, at least twice a week, reduced the risk of the early stage of memory loss, usually anticipating neurological diseases (173). Mushrooms enclose countless elements presently being under investigation in relation to brain health, particularly endogenous factors that control cell proliferation and differentiation in the nervous system (162).

Stimulators of nerve growth have been found in *Hericium erinaceus*. From this mushroom, several natural aromatic compounds, hericenones from the fruiting bodies and erinacines from mycelia, were isolated and studied as having potential beneficial effects in ameliorating cognitive functioning, and behavioral deficits in Alzheimer's and Parkinson's diseases (163, 174).

We have investigated the immune modulation role of mushroom biomass of *Coriolus versicolor* in neuroinflammation and neurohormesis and the emerging role of lipoxinA4 and inflammasome in progressive neurodegenerative disorders and distinct configurations of chronic mental illness, proposing a potentially innovative treatment (69).

**Figure 11:**
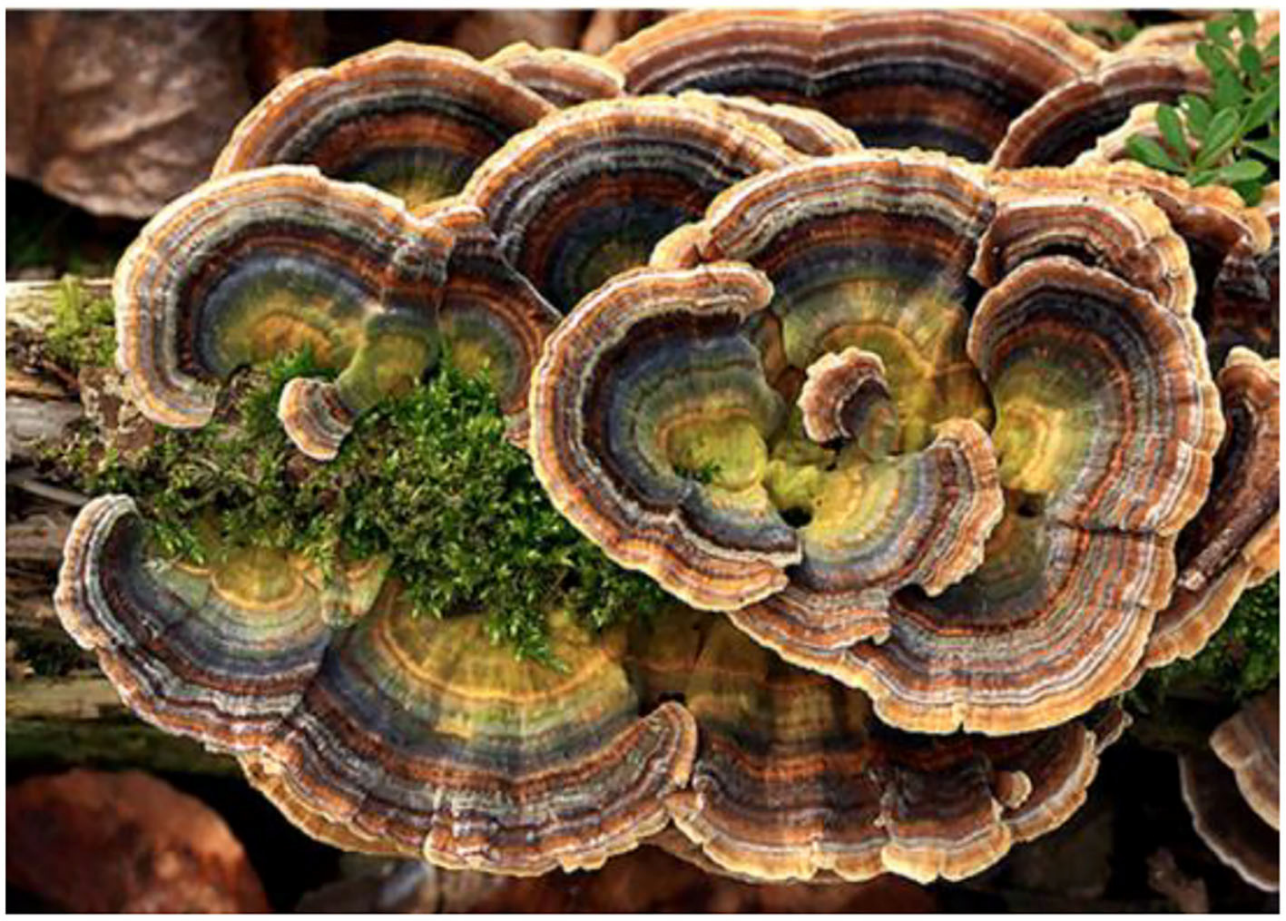
Edible, not eatable, *Coriolus versicolor* mushroom.

*Coriolus versicolor* (Figure 11) and *Hericium erinaceus* have been the subject of neurobiological research on the control of redox-dependent genes, so-called the vitagene family, measuring heat shock proteins, superoxide dismutase, glutathione and thioredoxin systems, lipoxin A4, and sirtuins, as potential neuro-therapeutic targets (165, 175).

Anxiety with overwhelming feelings of worry, nervousness, and fear, and depression with lingering low, sad, or hopeless mood are in developed countries among the first six most common primary care challenges in medical practice. Treatment with psilocybin, the psychedelic compound found in “magic mushrooms,” has shown promise in research settings for treating a range of mental health disorders and addictions (176).

Mushrooms (e.g., *Boletus badius*) and tea leaves contain the unique L-theanine amino acid, an analog of amino acids L-glutamate and L-glutamine, which may modulate aspects of brain function in humans. It showed multiple beneficial effects on depressive symptoms, anxiety, sleep disturbance, and cognitive impairments. Although the European Union does not support L-theanine as a supplement on health claims, it is endorsed by the FDA granted GRAS (generally recognized as safe) status by the Food and Drug Administration (167, 177).

## Autism spectrum disorder and mushrooms

Autism is also referred to as autism spectrum disorder (ASD) and constitutes a lifelong diverse group of conditions related to the development of the brain. It is characterized by some degree of difficulty with social and communication interaction affecting some 70 million people globally.

According to the World Health Organization, in 2022, one child in 100 worldwide suffers from an autism spectrum disorder (178). Through epidemiological estimates, these numbers have been increasing worldwide, especially in previously under-represented regions such as Africa and the Middle Eastern region; it is shown to be more common in Africa than initially believed, being a growing global public health concern.

Populations in developing countries are largely unaware of autism, diagnosed only around age 8, some 4 years later than worldwide, despite its prevalence universally similar, that is some 1% of the children. This neurological disorder is often hidden due to cultural, religious, and traditional barriers families and confounded with occult bewitching or profanity, ghosts, and devils.

Many children with this lifelong neurodevelopmental disability, more often boys than girls, in low-income neighborhoods are usually concealed indoors; therefore, the rate of occurrence is unrevealed, while scarcely any clinician has the know-how or experience to recognize the condition. Indeed, child mental health assistance has been minor, since child hunger, malnutrition, and mortality are considered the paramount concerns in developing countries.

Autism spectrum disorder is a congenital or environmental disorder caused by apoptosis, more than decreased neurogenesis, inducing significant hippocampal-dependent learning and memory impairment. Children with autism have a surplus of synapses in the brain, a slowdown of synaptic pruning, and novel and much-needed treatment may be based on reducing synapsis density (179).

The first FDA-approved psychedelic treatment, controlled substance esketamine, from hallucinogenic mushrooms is considered a major “breakthrough for depression.” Edible mushrooms (e.g., Chaga—*Inonotus obliquus*, Reishi—*Ganoderma lucidum*, Turkey Tail—*Coriolus versicolor*, and Shiitake—*Lentinula edodes)* are rich in ketamine and ergothioneine and have been successfully used on mental health highlighting the potential clinical and public health importance of mushroom consumption as a means of reducing depression and preventing other diseases (180, 181).

**Figure 12:**
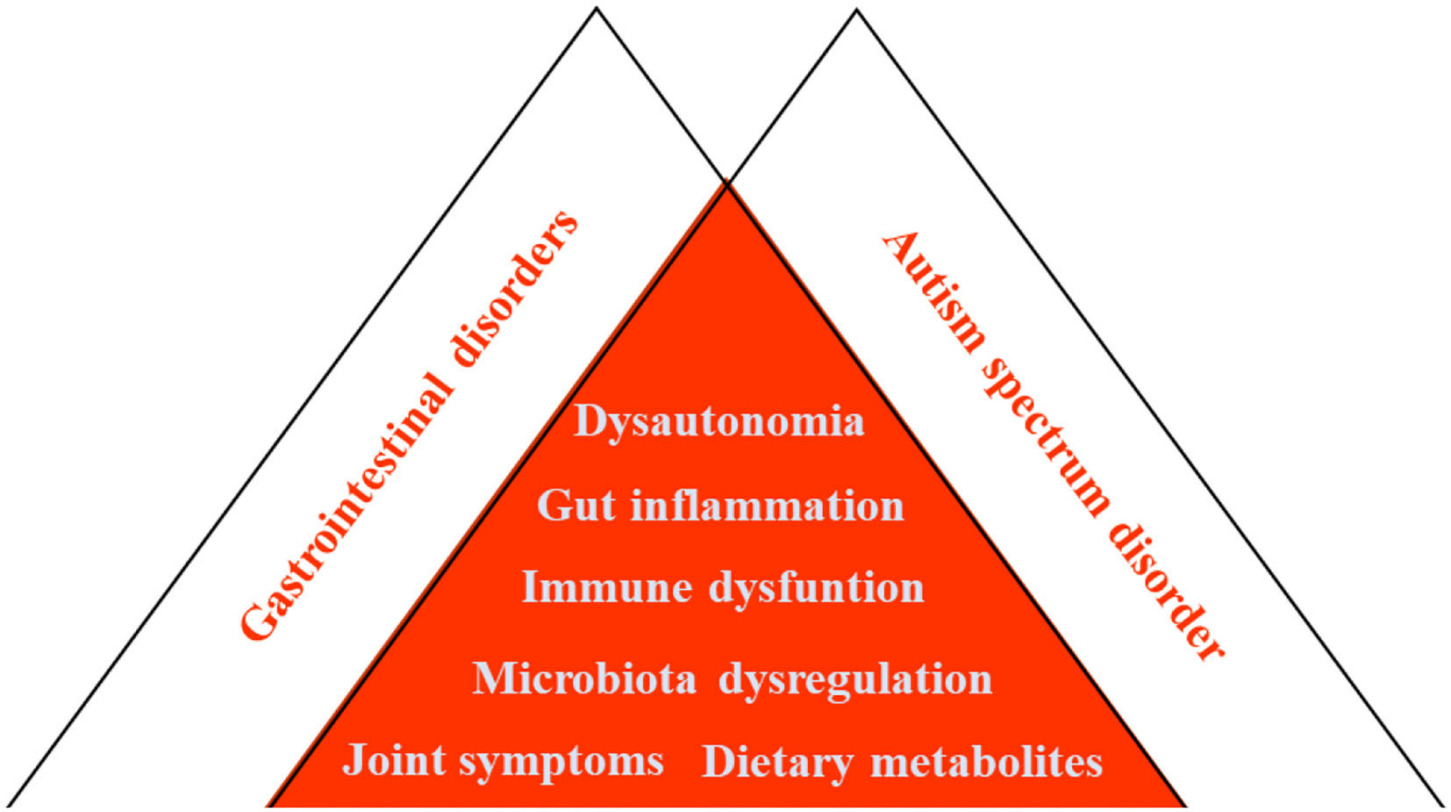
Concept of the overlap syndrome of GI disorders and ASD.

In this group of developmental disabilities, many, but not all patients with ASD suffer from gastrointestinal comorbidities, including acute and chronic constipation and diarrhea as well as persistent neuroinflammation (Figure 12). Managing an array of comorbidities greatly increases the complexity of managing disease, and it is important to address neuroinflammation by dietary restriction (182).

Preliminary results in the mouse colitis model suggest that *Coriolus versicolor* biomass supplements hold great promise as a useful way to manage inflammatory bowel diseases (183).

*Hericium erinaceus* various bioactive compounds are soluble, hence absorbed and metabolized, crossing the blood–brain barrier and clinically effective against depression. They condition several functions, promoting oligodendrocyte maturation with an increase in myelin basic protein, triggering the production of nerve growth factor (NGF) produced by local fibroblasts, a powerful neural survival factor after nerve injury or prolonged inflammation (174).

## Mushroom nutrition in Meniere's disease

Meniere's disease (MD) is a clinical syndrome affecting ~12 in every 1,000 people worldwide, and the underlying etiology of MD remains largely unknown. It is characterized by episodes of spontaneous dizzy spells (vertigo) associated with fluctuating, low-to-medium frequencies sensorineural hearing loss, tinnitus, and aural fullness in one or both ears. Meniere's disease can affect social life, productivity, and overall quality of life (184).

Increasing evidence suggests that, as an oxidant disorder, oxidative stress, immunomodulation, and neuroinflammation may be central to its pathogenesis. At present, there is no cure for this distressing neurodegenerative condition, but surgery and stem cell-based therapy have procedures with some success (185).

An open-label clinical trial in 40 patients with MD suffering from the cochlear sensorineural hearing loss was conducted where 22 of the patients were treated with *Coriolus versicolor* biomass (3 g/day for 2 months), and the remaining 18 were not treated. It was demonstrated that *Coriolus versicolor* supplementation may provide a useful means to modulate and amplify the body's response to oxidative challenge and cellular stress in Meniere's disease. This improved stress response appears to translate into measurable symptom relief, reduction in tinnitus, and improved mood (186).

## Concluding remarks

Natural products and structural analogs have historically made a major contribution to pharmacotherapy and are increasing the trust of western people for the treatment and management of several chronic diseases. Mushroom, as functional foods, nutraceuticals, or pharmanutrients are indeed regarded as next-generation functional foods, supporting health and wellness. Nevertheless, despite their very long history of use, safety profile, and clinical use, the differences among fresh mushrooms, their extracts, or biomass dietary supplements' effects on human health are still unidentified.

To aggravate the issue, the specificity of each type of mushroom with its high-molecular diversity and biological attributes, toward a particular health concern, still requires further evaluation namely at the identification of the responsible biomolecule and nanoparticle components as well as the dose response.

Furthermore, mushrooms may be safe as a vaccine adjuvant, but there is mild concern about using them to treat people with for example active SARS-CoV-2 infection since an immune-stimulating agent like mushroom might supercharge an individual's immune response, leading to a cytokine storm, posing the greater risk of COVID-19 mortality.

New efforts are needed to elucidate the still unknown bioactive compounds present in different mushrooms and their therapeutic potential. Novel toxicological studies are needed to ensure their safety and promote pre- and clinical studies.

## Author contributions

TF and VB conceived and wrote the article based on previous joint work. VB designed the figures. CS conducted part of the literature review. JG supplied African data and critical feedback. All authors contributed to the article and approved the submitted version.

## Funding

The present work was supported by CIISA for publication fees on behalf of FCT—Fundação para a Ciência e Tecnologia, Grant: UIDB/00276/2020.

## Conflict of interest

The authors declare that the research was conducted in the absence of any commercial or financial relationships that could be construed as a potential conflict of interest.

## Publisher's note

All claims expressed in this article are solely those of the authors and do not necessarily represent those of their affiliated organizations, or those of the publisher, the editors and the reviewers. Any product that may be evaluated in this article, or claim that may be made by its manufacturer, is not guaranteed or endorsed by the publisher.
